# Humoral biomarkers of latex allergy

**DOI:** 10.1186/2045-7022-4-S3-P128

**Published:** 2014-07-18

**Authors:** Pedro Giavina-Bianchi, Laila Sabino Garro, Marcelo Vivolo Aun, Antonio Abílio Motta, Mariana Castells, Jorge Kalil

**Affiliations:** 1 University of Sao Paulo, Clinical Immunology and Allergy, USA; 2 University of Sao Paulo, Clinical Immunology and Allergy, Brazil; 3 Harvard Medical School, Division of Rheumatology, Allergy and Immunology, Department of Medicine, BWH, USA

## Background

Although latex allergy is decreasing in several countries, it is still a global health problem that is associated with life-threatening reactions. The main objective of this study is to identify clinical and laboratory factors associated with sensitization and allergy to latex, including latex-specific IgE, IgG4 and IgA.

## Method

Observational study in a cohort of 400 children and adolescents with defect of neural tube closure. Patients underwent clinical interview and had their blood drawn to measure specific serum IgE, IgG4 and IgA to latex. The prevalence of sensitization and allergy to latex were calculated and possible associations with clinical and laboratory variables were analyzed.

## Results

The prevalence of sensitization and allergy to latex in patients presenting defects in neural tube closure was 33.2% and 12.2%, respectively. Cutaneous manifestations of latex allergy were the most common (79.6%), but anaphylaxis was observed in 4.75% of patients. Clinical and surgical factors associated with latex allergy were identified and a symptom score to screening patients was developed. Concentration of specific IgE to latex >0.77 kUA/l presented good accuracy in differentiating asymptomatic sensitization from allergy. Measurement of specific IgE to recombinant allergens also showed good accuracy in the diagnosis of allergy. The specific serum IgG4 concentration was negatively associated with allergy to latex, but this was not observed for specific IgA.

## Conclusion

Higher concentration of specific IgE to latex and Hevb5, lower concentration of specific IgG4 to latex and symptom score ¡Ý 40% were associated with latex allergy.

